# Chronic Abdominal Pain Secondary to Mesh Erosion Into Ceacum Following Incisional Hernia Repair: A Case Report and Literature Review

**DOI:** 10.14740/jocmr1730w

**Published:** 2014-02-06

**Authors:** Fahad Aziz, Misbah Zaeem

**Affiliations:** a Penn State Hershey Medical Center, 500 University Drive, MC, Hershey, PA 17033, USA; b Wake Forest School of Medicine, Winston-Salem, NC, USA

**Keywords:** Hernioplasty complication, Mesh migration, Ceacum erosion, Chronic abdominal pain

## Abstract

Incisional hernias following abdominal operations are a common complication. Mesh is frequently employed in repair of these hernias. Mesh migration is an infrequent occurrence. We present the case of transmural mesh migration from the abdominal wall into the ceacum presenting as chronic abdominal pain. Given the popularity of minimally invasive surgery utilizing polypropylene mesh for incisional hernia repair, related complications such as postoperative hematoma and seroma, foreign body reaction, organ injury, infection, mesh rejection and fistula are increasingly being noted. Most of the mesh migrations reported in the literature involve the urinary bladder. We present a case of delayed mesh migration into the ceacum. Mesh migration is a rare and peculiar complication that is rarely reported in the literature. A review of the literature shows that there are no other cases of mesh migration into ceacum several years after open type incisional hernia repair.

## Introduction

Incisional hernia is the most common complication of abdominal surgery, with incidence up to 10-15% and recurrence rates of 20-45% [1, 2]. These hernias are often repaired with synthetic mesh to reinforce the repair or to reduce tension on weakened or missing abdominal wall fascia. This case represents one unusual complication of using mesh. Mesh migration is an infrequent occurrence. The offending lesion was found to be the prolene mesh having eroded into the ceacum. The migration of mesh into the ceacum was likely the result of several unusual events: polypropylene stitches migrating through the abdominal wall, mesh freely moving in the abdominal cavity and complete erosion through the ceacum into the lumen. Technical factors as well as patient factors are likely involved in these cases. Due to the rare nature of these events, it is unclear whether type of mesh or type of fixation played a role in mesh migration into bowel. Mesh migration has rarely been reported.

## Case Report

The patient is a 56-year-old female with a past medical history of hypertension and fibroids. She had multiple abdominal surgeries including two cesarean sections and total abdominal hysterectomy. Five months later after her last surgery, the patient presented with a large incisional abdominal hernia. She had hernia repair with polypropylene mesh under general anesthesia. Her postoperative course was uncomplicated.

**Figure 1:**
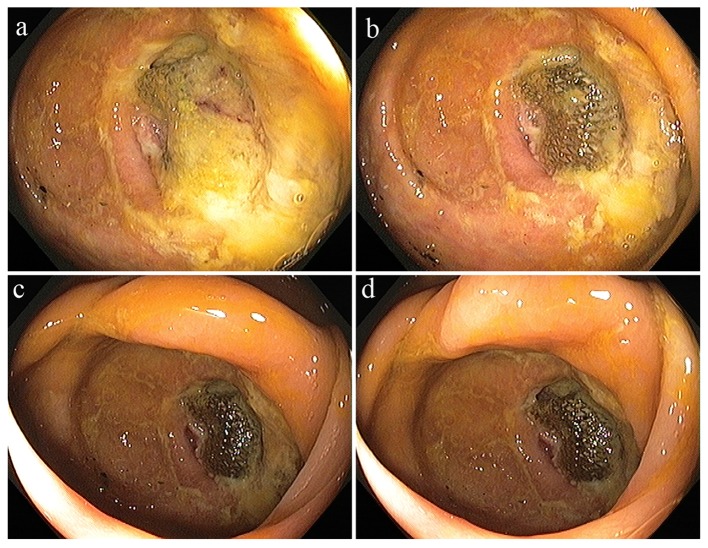
(a-d) Diffuse diverticulosis and a foreign body in the cecum.

Approximately 14 years after hernia repair, the patient presented to the emergency department with the complaints of intermittent, abdominal pain, starting at right lower quadrant and migrating upwards to all over abdomen, associated with episodes of nausea. She had this pain from the 2 years prior to the presentation, which had been getting worse. She denied any other complaints. She had good bowel sounds on the physical examination. All lab work was unremarkable. Computed tomography (CT) scan of abdomen and pelvis was also unremarkable. She got colonoscopy as part of regular screening process which showed diffuse diverticulosis and a foreign body in the cecum (Fig. 1). In light of the patient’s past history of incisional hernia repair, the foreign body was consistent with migration of previous surgical mesh into the ceacum.

## Discussion

The use of metallic mesh to reinforce the approximated tissues of a hernia repair or to actually replace the defect developed widespread use by 1946. An increase in wound complications, such as serum accumulations, wound infections and persistent draining sinuses resulted from the use of tantalum or stainless steel mesh. Consequently, the use of tantalum or stainless steel mesh, whole skin and cutis was completely abandoned by 1970 [2]. A knitted polypropylene mesh (Marlex) introduced in 1959 and expanded polytetra fluoro-ethylene patch (Gortex) have been increasingly used in hernia repairs over the past three decades. Use of fascia lata, while still an excellent alternative to synthetic material in specific cases, has significantly declined in hernia repairs.

Mesh repairs minimize the amount of tension that must be put on the abdominal wall in order to cover the hernia, and are generally considered preferable for incisional hernias. In a long-term retrospective study from Europe, the incidence of recurrence of incisional hernias after simple sutured repair was over 60%; the use of mesh decreased the recurrence rate to approximately 30% [3-5]. Mesh repair is particularly important for incisional hernias with a diameter greater than 4 cm as the risk of recurrence is likely related to the tension placed on the repair in large hernias. Since then, many types of mesh have been developed. Complications related to the use of artificial materials in hernia repair include postoperative hematoma and seroma, foreign body reaction, organ injury, infection, mesh rejection and fistula. Mesh migration following hernia repair is an uncommon complication. Erosion into a viscous can be associated with migration or can occur with the mesh in the intended position.

When this occurs, infection, abscess, fistula, or obstructions are the most common sequelae. Migration to a completely intra-luminal position is exceedingly rare. Mesh migration occurs generally through two mechanisms. Primary mechanical migration occurs when an inadequately secured mesh traverses along adjoining paths of least resistance or when a relatively secure mesh is displaced by external forces [1]. Secondary migration, on the other hand, occurs through trans-anatomical planes and is the result of erosions triggered by foreign body reaction [6, 7]. This mechanism has been supported by the presence of inflammatory granulation tissue at the site of migration. The latter process is gradual and may take several years [8].

Mesh migration is rare and unpredictable. Clinical presentations are variable and related to the organ involved. Migration of mesh into the urinary bladder has been reported to cause hematuria and recurrent urinary tract infections [9]. One report noted mesh migration into the scrotum after laparoscopic hernia repair that presented as a tender scrotal mass [10]. In another report of scrotal migration of mesh, strangulating bowel obstruction was the presenting feature [11]. Several reports of resultant enteric and enterovesical fistulas have been reported [12-14]. More ominous findings such as bowel obstruction and sigmoid perforation have also been documented [15-17]. Successful colonoscopic removal of a migrated mesh from the colon at the splenic flexure has also been reported. After a literature review discussing the significant complications that result from mesh migration, the authors hypothesize that the method of fixation, as well as type of mesh, may have contributed to this problem.

The method of fixation may affect migration rates by altering the tensile strength and degree of movement of the mesh. The nature of the biomaterial is also important, as it affects the extent and degree of interaction with the surrounding tissue [18]. The size, shape and positioning of the mesh may also be significant. One study, in particular, showed that new low-weight mesh shows significantly less cellular proliferation and foreign body reaction than traditional prolene mesh. Biologic agents are being used with increasing frequency in abdominal wall hernias, where they have been shown to decrease foreign body reaction and potential infectious complications. There are one to two case reports of mesh migration into small bowel, bladder, large bowel, ceacum, but all after laparoscopic inguinal hernia and this is first case after open incisional hernia.

### Conclusion

In conclusion, mesh migration, particularly erosion, is a rare complication of any incisional hernia repair, especially following total extraperitoneal repair. There is no clear cause of this complication, but new methods of mesh fixation, as well as types of mesh, are being investigated. It should also be recognized that mesh complications, particularly erosion, tend to occur years later and should be considered in atypical patient presentations. If tissue is available to place between the mesh and bowel so that they are not in direct contact, this might help avoid this complication.

Given the popularity of these surgical procedures, complications may be frequently encountered; gastroenterologists should thus be aware of the potential complications and the appropriate management.
